# Thematic analysis of national online narratives on regular asymptomatic testing for Covid-19 in schools in England

**DOI:** 10.1186/s12889-023-15991-3

**Published:** 2023-05-31

**Authors:** Jo Taylor-Egbeyemi, Holly Carter, Charlotte Robin

**Affiliations:** Behavioural Science and Insights Unit, UK Health Security Agency, London, UK

**Keywords:** COVID-19, Public health, Asymptomatic, Lateral flow tests, Schools, Social media

## Abstract

**Objective:**

To understand the public perceptions of the schools Covid-19 testing programme in England.

**Design:**

Qualitative social media analysis.

**Setting:**

Online users of parenting forums (Mumsnet and Netmums), Facebook newspaper pages and Daily Mail online readers, who responded to posts or articles about the schools testing programme in England, between 1 and 31 March, 2021.

**Results:**

Overall, seven main themes were identified, these were divided into barriers and facilitators to engaging in testing for Covid-19. Barriers were: uncertainty around testing in the absence of symptoms; concerns about testing; implications about testing positive; mistrust in the Government. Facilitators were: desire to protect others; desire to return to normality; and hearing others’ positive experiences.

**Conclusions:**

Our analysis highlighted that alongside well-established barriers to engaging in asymptomatic testing, parents were having to negotiate additional complex decisions around balancing their child’s anxiety over testing alongside acknowledgement of the implications of regular testing, such as return to normality and protecting others. Parents and children would benefit from additional practical and social support to facilitate engagement with the schools testing programme.

## Strengths and limitations of this study

- Analysis of publicly available data from parenting forums and other social media sources is a useful way of rapidly assessing public narratives on the schools testing programme, particularly from people who may not usually engage with surveys or interviews.
- We sampled from a range of different social media platforms, to account for the differences in demographic characteristics of social media users. However, we were limited to Facebook, parenting forums and Daily mail as other online newspapers we identified had disabled comments on Covid-19 articles.
- To uphold anonymity of users, we did not seek to obtain information from user profiles or accounts therefore we have no information on whether data were from parents, teachers or members of the general public.
- It was not possible to identify location of the person writing the comment, however as the schools testing programme was only implemented in England it is less likely that people from outside England would respond to information about the testing programme as it was less relevant to them.
- Future research could seek to include a wider range of online media sources aimed at school children as well as parents.

## Background

The Covid-19 pandemic started in late 2019 and spread globally. The first Covid-19 cases in the UK were identified on January 31, 2020 [1]. The initial response to COVID-19 in the UK was focused on containment measures, which were in place from January to March 2020. During this time, the government took measures to prevent the spread of the virus, including implementing travel restrictions and isolating individuals who had recently travelled to areas with high infection rates. However, in the absence of widespread testing capabilities at the time, it was difficult to accurately identify and track the spread of the virus [2]. Lateral flow tests were approved for home use and schools in December 2020 [3].

To help mitigate community transmission of Covid-19 in England during the pandemic, several control policies were put in place. One such policy was the closure of schools with schools being closed from March 2020 until June 2020, and then again from January 2021 until March 2021 [4]. While Covid-19 appears to cause a relatively mild illness in children and mortality rates in this population are low [5], there is uncertainty around the role of children in Covid-19 transmission. Previous research has highlighted that children can spread viruses more easily than adults and are less likely to adhere to protective behaviours, such as handwashing [6]. With Covid-19, one of the biggest concerns is the transmission of the virus to contacts of school children, particularly older adults or vulnerable people in their households or bubbles [7]. Between April and May 2021, there were 97 Covid-19 outbreaks in English schools [8] and surveillance has shown that there are more outbreaks in secondary schools compared with primary schools [9]. As schools were closed at the start of the pandemic, it is difficult to say whether children are at higher risk of transmission in school settings compared with community settings [10].

While school closures may have helped reduce community transmission of the virus [11], concerns about the risks of transmission have been outweighed by worries about missed learning and child welfare, particularly among those from vulnerable backgrounds [12]. When students are not receiving face-to-face education, the lack of routine that school usually provides for students has exacerbated existing mental health issues [13]. This risk has been higher in those with special educational needs, due to the disruption in their routine [13].

When schools reopened, several non-pharmaceutical interventions (NPIs) were implemented to reduce transmission [14] including physical distancing measures, staggered drop-off/collection times, wearing of face coverings, hand hygiene and the introduction of school “bubbles”. One study highlighted that more than half of parents felt positive about their children returning to school, with 82% of parents at least partly reassured by the Covid-19 preventive measures implemented, although a third of parents also reported feeling some anxiety [15]. In March 2021, when schools reopened again in England, routine asymptomatic testing using rapid lateral flow tests (LFTs) was introduced for staff and students. Staff and students at secondary schools were tested on-site and were also provided with two tests each week to use at home. Households and bubbles with school age children were also required to conduct tests twice per week [16]. Primary school children were not required to test.

While regular asymptomatic testing is not currently required in schools, it is likely that similar forms of testing will be required to manage future outbreaks of Covid-19 or other respiratory viruses. Previous research has highlighted that regular testing of school age children has the potential to control within-school and community transmission [7, 17], and could therefore play a key role in reducing disease transmission in these settings. However, the effectiveness of regular testing in schools to reduce transmission relies on engagement from parents and students, and high uptake with the testing programme. Previous research has identified barriers to regular asymptomatic testing in different contexts, which may have a negative impact on the effectiveness of the testing programme. Such barriers include concerns about stigma of testing positive [12]; a low perceived risk from the virus [18]; and mistrust in the Government [19]. Facilitators of engagement with asymptomatic testing include a sense of community [20]; wanting to protect others; a desire to return to normal [21]; and perceived efficacy for reducing asymptomatic transmission [18].

While research has highlighted barriers and facilitators to regular asymptomatic testing in other contexts [18, 19], there is a need to understand barriers and facilitators to engagement with regular asymptomatic testing in schools. To address this, we carried out rapid thematic analysis of social media data and online newspaper comments to understand public perceptions of asymptomatic testing in schools. Social media is becoming an increasingly common source of gathering data on behavioural insights [18, 19] and how populations react to public health strategies, including mass asymptomatic testing. In recent years there has been an increase in user-generated online content [20], including an increase in the use of comments sections on online newspapers [21]. Readers commenting on the news via these routes is one of the most common forms of citizen engagement with the news [20] and analysing these comments can be a pragmatic method to give an overall impression of ongoing conversations that are occurring in response to news articles [21]. The aim of this study was to use an analysis of social media data to understand public perceptions of asymptomatic testing in schools in England, particularly from parents who made the decisions about their child engaging with testing. We wanted to identify barriers and facilitators to engaging in the schools testing programme, to increase uptake and ensure engagement with regular asymptomatic testing in schools.

## Methods

### Data sources and sampling

Data were collected through the following publicly accessible online sources: parenting forums (Mumsnet and Netmums); national newspaper Facebook pages; and comments sections from an online national newspaper (the Daily Mail), between 1st and 31st March 2021. This time period covered prior to the school testing starting and the start of the testing period. We sampled in the week before testing was implemented and the first few weeks of testing so we could gather responses to the announcement of the testing programme and any experiences of taking part. The same keyword search was used for each data source: *school, nursery, college, student, pupil, covid-19, testing, asymptomatic, lateral flow test, re-opening, mass testing, home tests, LFD.*

#### Parenting forums

Mumsnet is the largest parenting forum in the UK, with over 6 million unique visitors each month [22], whereas Netmums has around 1 million members [23]. Both forums were included as the demographic characteristics of members are different; Mumsnet users are slightly older, more likely to have completed higher education [22] and with a larger proportion of users with a household income over the national average [23]. Netmums has around 30% of members with above average incomes and 40% from low-income families [23].

Mumsnet and Netmums were searched for relevant posts about schools testing, between 1 and 31 March, using the keyword search (detailed above). Posts were summarised by source, title, date and number of replies. The four posts in each forum with the most replies were included in the study; eight posts with a total of 995 replies (Table 1).

**Table 1:** Details of data sources and number of comments sampled 1–31 March 2021

Data source		Number of comments
Daily Mail comments^a^		800
Online newspaper Facebook pages^b^	Daily Express Daily Mail The Mirror The Metro The Independent The Guardian The Sun The Telegraph	36 97 100 100 100 100 100 100
Parenting forums^c^	Mumsnet Netmums	966 29
	Total number of comments	2528

^a^ The Guardian and Daily Mail are the most widely read digital newspapers [24]. The Guardian had disabled their comment function at the time of data sampling. Eight Daily Mail articles were identified and the first 100 comments from each article were included in the analysis

^b^ In 2020, 45% of people reported they consume their news via social media, mostly Facebook (75%) [24]. The article with the most comments on each newspaper Facebook page was sampled, up to a maximum of 100 comments per article

^c^ Mumsnet is the largest parenting forum in the UK, with over 6 million unique visitors each month [22], whereas Netmums has around 1 million members [23]. Both forums were included as the demographic characteristics of members are different; Mumsnet users are slightly older, more likely to have completed higher education [22] and with a larger proportion of users with a household income over the national average. Netmums has around 30% of members with above average incomes and 40% from low-income families [23]

#### Newspaper comments

The Guardian and Daily Mail are the most widely read digital newspapers in the UK [24], however the Guardian had disabled their comment function at the time of data sampling, therefore newspaper comments were sampled from the Daily Mail.

All articles in the Daily Mail relating to schools testing (posted between 1 and 31 March 2021) from these sources were identified using the keyword search (detailed above). Articles were summarised by source, title, date and number of comments. The eight Daily Mail articles with the most comments were identified and the first 100 comments from each article were included in the analysis; 800 comments in total (Table 1).

#### Facebook posts

In 2020, 45% of people reported they consume their news via social media, mostly Facebook (75%) [24]. Facebook pages for the most popular newspapers were included in the study: Daily Express, Daily Mail, The Mirror, The Metro, The Independent, The Guardian, The Sun and The Telegraph. Each Facebook page was searched using the keyword search (detailed above) for articles posted between 1 and 31 March 2021. The article with the most comments on each newspaper Facebook page was sampled, up to a maximum of 100 comments per article; 733 comments in total (Table 1).

All publicly accessible comments on identified posts or articles were copied and pasted to text documents for coding. A total of 2528 comments from all three data sources were analysed, details of data sources and sampling are shown in Table 1.

In line with British Psychological Society (BPS) guidelines [25] for conducting internet-mediated research, this research did not require ethical approval because only publicly available data (comments posted in response to public Facebook posts, online forums, or comments posted in relation to online media articles) were used.

### Data analysis

Data were depersonalised by removing any identifiable data (including names and locations) and imported into NVivo for analysis. An inductive approach using open coding [26] on approximately 25% of the data was used to develop the initial coding framework, by identifying key themes of interest. During this stage, 10% of the data were coded independently by a second coder and meetings were held to discuss coding and reach a consensus on the coding framework. This framework was applied to the remaining data and additional codes were developed as the analysis progressed. Analysis was conducted by a UKHSA employee therefore reflexivity was exercised by taking into account possible influences of their position as a government employee on the analysis.

## Results

Seven key themes and ten subthemes were identified. Themes could be broadly grouped into barriers and facilitators to engaging with school testing. Themes relating to barriers to testing were: uncertainty around testing in the absence of symptoms; concerns about testing (testing induced anxiety,, children with existing health conditions, ability to conduct the test properly,, accuracy and safety of tests); implications about testing positive (financial implications, feeling blamed); and mistrust in the Government (perception of testing as a way to maintain fear; perception of testing as a form of abuse). Themes relating to facilitators of testing were: desire to protect others (protecting family, protecting wider community); desire to return to normality; and hearing others’ positive experiences. A description of each theme and sub-theme are included in Table 2, along with additional example quotes.

**Table 2:** Overview of themes and sub-themes identified in the analysis of social media data on schools testing in England

Theme	Sub-theme	Description	Example quotes
Barriers to engaging in testing			
Uncertainty around testing in the absence of symptoms		The belief that people should not have to take a test if they are not experiencing symptoms.	*Wow the world has gone mad, testing healthy people/kids to prove they are ill.* The Metro, 25.03.2021 *Perhaps we should screen them for any and all diseases known and unknown to man because there is a risk that one of them could be carrying a disease???* The Daily Mail, 08.03.2021
Concerns about testing	Testing induced anxiety	Some parents believed that taking a test would cause their child anxiety. In some cases, it was the parents who were experiencing anxiety in place of their child.	*My son is having a panic as he’s just found out that he will have to have a COVID test at school. His worry is that taking a throat swab will make him gag and he has a real fear of being sick.* Mumsnet, 03.03.2021 *In fact we have had more issues the other way - the child wants to be tested but the parents haven’t sent the consent in.* Mumsnet, 03.03.2021
	*Children with existing health conditions*		*“I haven’t given consent for my children to be tested for the same reason. They both have Aspergers and both have said they don’t want to do the throat swab” (Mumsnet, 03.03.2021).* *“My daughters got autism and very worried about having this test” (The Mirror, 07.03.2021)* *“SEN guidelines to her school it is not mandatory and if a child shows distress in anyway they can NOT proceed. My son won’t even have his temperature taken unless it’s a non-touch thermometer so a test would send him into an absolute meltdown and people would get hurt” (The Mirror, 07.03.2021)*
	Ability to conduct the test properly	The belief that children would struggle to conduct the test properly themselves, which could lead to false positive or false negative results.	*Surely it depends if the test is done properly. I would imagine it is VERY accurate if done properly, but if you don’t want a positive Covid result you barely scrape, or get near, the throat or nose. Herein lies the problem surely.* Daily Mail, 24.03.2021 *“My worry is that these kids won’t do the test right and then we’ll be getting false results and sending these kids to school symptoms they think is unrelated to covid due to negative test results. My daughter had 2 negative covid tests with all the symptoms I just don’t trust this at all” (*The Mirror, 07.03.2021).
	Accuracy and safety of the tests	Anxiety about the accuracy of the tests in general, particularly around false positive results and the impact that would have on children’s education.	*The tests return so many false positives … I feel for the kids as they are not even affected.* Daily Express, 08.03.2021 *“1 in 3 Lateral Flow tests are false & we all know the PCR tests are not fit for purpose*” (The Mirror, 07.03.2021)
Implications about testing positive	Financial implications	Concern over the financial implications on the parents when a child tested positive. This was exacerbated by the belief the tests were not accurate.	*The tests are not even reliable! Some of us haven’t got the time or money to sit on our bums all day self-isolating for 10/14 days because of a rubbish test.* Daily Mail, 08.03.2021
	Feeling blamed	Parents expressed feeling blame for their child’s positive test. In some cases, these feelings of blame were reinforced by official messaging; those positive cases were a result of not following guidance. In some cases, there was also concern that the schools testing policy would result in children being blamed for further lockdowns.	*Did you do a rapid test before they went back or is it that they’re blaming you because you didn’t bother?* Mumsnet, 10.03.2021 *Unfortunately, a lot of information that was initially stated was that it was only people who broke the rules who caught Covid, or its only caught by people’s selfish behaviour etc. etc. So many people have clung onto this idea that they then blame others for simply getting a highly contagious disease.* Mumsnet 10.03.2021 *No doubt the kids will get the blame for the already planned lockdown*. The Mirror, 07.03.2021
Mistrust in the Government	Perception of testing as a way to maintain fear	The belief that the Government and media are using testing as a tool to control public behaviour through maintaining fear about COVID-19 transmission.	*That was always the point… Test millions of school children every week, to inflate the number of ‘cases’ and maintain fear!* The Telegraph, 31.03.2021
	Perception of testing as a form of abuse	Equating regular asymptomatic testing of children to child abuse, including the belief children were being manipulated into the normalisation of taking regular tests.	*I am a lawyer, this is abuse, battery, assault whatever you want to call it. Plain and simple, I would never have agreed to anything like this. My daughter is too important, and I’d never let someone assault her twice a week.* Daily Mail, 06.03.2021 *When they think they have to take a test to see if they can go to school or do they have to go home because they have a disease and could kill people, it seems to me these poor children are being groomed by the Government and their scientists for this new normal, I feel very sorry for them.* The Mirror, 07.03.2021
Facilitators to engaging in testing			
Desire to protect others	Protecting family	The view that regular testing of children was a way to protect children and the rest of the family from COVID-19.	*It makes sense doesn’t it for the whole family’s piece of mind, at least you’re doing the right thing.* The Mirror, 07.03.2021
	Protecting the wider community	The view that regular testing of children would help protect the wider community. This included teaching children social responsibility.	*Absolutely. I don’t understand why people wouldn’t be happy to know their child and other people’s safety is what matters here.* The Mirror, 07.03.2021 *My kids (many with special needs and managing tests no problem) and teachers and carers have all benefitted so that’s good enough for me* The Guardian, 06.03.2021 *You ask your children to do it protect other children and teachers from catching it from them, it’s called social responsibility.* Mumsnet, 06.03.2021 *The messages you send to your child when you refuse the get them tested*: *You think they can’t handle it (the vast majority of them can and you should teach them you believe they can)* *You teach them that social responsibility and personal responsibility is not important (it is)* *You teach them that you can protect them always from things they will find tough (you can’t)* *You teach them their teachers are not worth protecting (they are).* Mumsnet, 03.03.2021
Desire to return to normality		The belief that regularly testing children would help with a return to normal life, particularly enabling children to return to school for face—to-face lessons.	*Really hope this plan works… it will be awful to have sick children and another lockdown.* Daily Express, 06.03.2021 *Children need to be back in school. Happy for my daughter to be tested, they need normality back in their young lives.* Daily Mail, 06.03.2021
Hearing others’ positive experiences		Sharing of positive experiences of testing helped reduce the concerns of other parents and encouraged them to consent to their children being tested.	*Pleasantly surprised by how much of a non-event it’s all been.* Mumsnet, 03.03.2021 *He is very much calmer - as am I - so thank you all - this thread has been very helpful.* Mumsnet, 03.03.2021

### Barriers to engaging in testing

#### Uncertainty around testing in the absence of symptoms

For some people, the absence of symptoms negated the need for testing. In some cases, this acted as a barrier to testing and there was resistance to the notion of being tested for an illness that neither they nor their child were experiencing symptoms of. The absence of symptoms meant they were healthy and did not need testing: “*Wow the world has gone mad, testing healthy people/kids to prove they are ill.”* (The Metro, 25.03.2021).

*“Perhaps we should screen them for any and all diseases known and unknown to man because there is a risk that one of them could be carrying a disease??? “(*Daily Mail, 08.03.2021).

### Concerns about testing

#### Testing induced anxiety

Parents expressed several worries about different aspects of the testing process, which acted as barriers to consenting to their child being tested. One such issue related to the anxiety testing may cause: “*My son is having a panic as he’s just found out that he will have to have a Covid test at school. His worry is that taking a throat swab will make him gag and he has a real fear of being sick.” (*Mumsnet, 03.03.2021).

School supposed to be an important part of a child’s life, this will cause a lot of stress, anxiety and a lot of upset kids: *“I’d happily do it to myself but no I wouldn’t put my young kids through it again unless they show symptoms. It’s not “fine” at all for them.” (*The Mirror. 07/03/2021).

In some cases, parents did not want their child to be tested, whereas the children themselves were happy to do the test: “*In fact we have had more issues the other way - the child wants to be tested but the parents haven’t sent the consent in.”* (Mumsnet, 03.03.2021).

#### Children with existing health conditions

For some parents, concerns about testing were exacerbated because their child had an existing health condition: *“I haven’t given consent for my children to be tested for the same reason. They both have Aspergers and both have said they don’t want to do the throat swab” (*Mumsnet, 03.03.2021).

#### Ability to conduct the test properly

An additional barrier to the testing in schools was the concern about conducting the tests properly. There were some conflicting opinions regarding how to conduct the tests and concern that if they were not conducted properly, then it would provide an inaccurate result: “*Surely it depends if the test is done properly. I would imagine it is VERY accurate if done properly, but if you don’t want a positive Covid result you barely scrape, or get near, the throat or nose. Herein lies the problem surely.”* (Daily Mail, 24.03.2021).

#### Accuracy and safety of the tests

For some, the accuracy of the tests was an additional barrier and some parents discussed having to navigate issues with false positives and the impact on their child’s education and balancing that with false negatives and the risk to their child’s (and other’s) health. There was concern that tests could return both false positives and false negatives: “*The tests return so many false positives … I feel for the kids as they are not even affected”* (Daily Express, 08.03.2021).

### Implications about testing positive

The impact of testing positive was highlighted as a key barrier. In some cases, this was because of the financial implications for the rest of the family if a child tested positive. In addition, there were concerns around children or parents being blamed for other children in their class or bubble being sent home to isolate.

#### Financial implications

The financial implications on the parents of a child testing positive were identified as a key barrier: “*The tests are not even reliable! Some of us haven’t got the time or money to sit on our bums all day self-isolating for 10/14 days because of a rubbish test.” (*Daily Mail, 08.03.2021).

#### Feeling blamed

Some parents felt that other parents would be angry with them or their child if the child tested positive, particularly given how potentially disruptive the implications would be for other children in the same school bubble. They felt that other parents may feel that they could have done more to avoid their child testing positive. In response to a thread in which one parent reported feeling blamed by other parents when their child tested positive, one person wrote: “*Did you do a rapid test before they went back or is it that they’re blaming you because you didn’t bother?” (*Mumsnet, 10.03.2021).

For some, there was also a feeling that official messaging embedded these narratives of blame; the only reason someone can test positive is because they have done something wrong, in this case not consenting to their child being tested: “*Unfortunately a lot of information that was initially stated was that it was only people who broke the rules who caught Covid, or its only caught by people’s selfish behaviour etc. etc. So many people have clung onto this idea that they then blame others for simply getting a highly contagious disease.*” (Mumsnet, 10.03.2021).

In addition to individual blame, for some there was a concern that if there was an increase in positive cases, then children would get the blame for any further lockdowns or restrictions: “*No doubt the kids will get the blame for the already planned lockdown*.” (The Mirror, 07.03.2021).

#### Mistrust in the government

Mistrust in the Government and its policies resulted in some people believing regular testing in schools was being used to maintain fear and therefore control behaviour. This led to criticism of policies and in some extreme cases, the schools testing programme was likened to harm or abuse of children.

#### Perception of testing as a way to maintain fear

A key theme was that people felt the Government and the media were maintaining fear of contracting the virus and using this to control the public’s behaviour. Some of the comments reflected the belief that the purpose for schools testing was to deliberately increase the number of positive cases, so that restrictions would be tightened again: “*That was always the point… Test millions of school children every week, to inflate the number of ‘cases’ and maintain fear*!” (The Telegraph, 31.03.2021).

*“I really hope parents refuse consent for testing and that children do not wear masks in class as there is no legal requirement to. Covid is nothing but fear-based lies used to create mass hysteria to enslave the population*” (Daily Mail, 05.03, 2021).

#### Perception of testing as a form of abuse

In some cases, the way in which schools testing was conceptualised by parents equated testing children to abuse. In addition, the language used was particularly strong, highlighting how strong some parents’ reactions to the policy were: “*I am a lawyer, this is abuse, battery, assault whatever you want to call it. Plain and simple, I would never have agreed to anything like this. My daughter is too important, and I’d never let someone assault her twice a week.”* (Daily Mail, 06.03.2021).

Highly emotive language was used when describing the Government and their motives for testing school children: “*When they think they have to take a test to see if they can go to school or do they have to go home because they have a disease and could kill people, it seems to me these poor children are being groomed by the Government and their scientists for this new normal, I feel very sorry for them.*” (The Mirror, 07.03.2021).

### Facilitators to engaging in testing

We identified the key facilitators to regular testing in schools as: the desire to protect others (protecting family, protecting wider community); the desire to return to normality; and hearing others’ positive experiences.

### Desire to protect others

#### Protecting family

For some, testing in the absence of symptoms provided reassurance that they were helping keep their child and other members of the family safe. Regular testing was viewed as an important part of keeping children safe: “*It makes sense doesn’t it for the whole family’s piece of mind, at least you’re doing the right thing.”* (The Mirror, 07.03.2021).

#### Protecting the wider community

In addition to regular testing being a way of keeping family safe, some people discussed the importance of testing children to protect the wider community: “*Absolutely. I don’t understand why people wouldn’t be happy to know their child and other people’s safety is what matters here.”* (The Mirror, 07.03.2021).

Some parents also felt that encouraging their children to test regularly taught them important lessons about responsibility to others in their community. As such, for these parents regular testing became a social responsibility. For these parents, there was a feeling that withholding consent to test would have wider implications on their children, beyond risk of Covid-19 transmission: “*The messages you send to your child when you refuse the get them tested: You think they can’t handle it (the vast majority of them can and you should teach them you believe they can). You teach them that social responsibility and personal responsibility is not important (it is). You teach them that you can protect them always from things they will find tough (you can’t). You teach them their teachers are not worth protecting (they are).”* (Mumsnet, 03.03.2021).

#### Desire to return to normality

There was an expectation that regular testing would be a step towards education returning to normal. For some, the desire for education to return to normality, including having their child back in school, was a key facilitator to testing. There was an awareness of the importance of education and while there were some concerns around the efficacy of the testing programme, the desire to return to face-to-face lessons encouraged parents to engage with regular testing: “*Children need to be back in school. Happy for my daughter to be tested, they need normality back in their young lives.”* (Daily Mail, 06.03.2021).

#### Hearing others’ positive experiences

When parents shared positive experiences of testing within the school community, this acted as a facilitator for others to engage in testing: “*Pleasantly surprised by how much of a non-event it’s all been.*” (Mumsnet, 03.03.2021).

This was particularly evident where parents had previously expressed concerns about the process of swabbing and testing their child: “*He is very much calmer - as am I - so thank you all - this thread has been very helpful.”* (Mumsnet 03.03.2021).

## Discussion

The purpose of this study was to understand the attitudes towards asymptomatic testing in schools in England by analysing social and online media data. It is important for perceptions to be assessed at the start of a public health response and at the time of introduction of a new policy as this is important and useful information for the next time a new policy is implemented (either for the next pandemic or for other public health responses).

The study also aimed to identify the barriers and facilitators to engaging in regular testing. Where concerns and barriers were identified, these were primarily discussed within the context of balancing children’s mental health and wellbeing with creating and maintaining a safe school environment. For some parents, it was deemed important for the wellbeing of their children to be taught within the school environment where possible, and if regular testing can facilitate this then it was seen as more acceptable. Several barriers were identified, and we provide some recommendations for how these could be addressed. The key barriers we identified were: uncertainty around testing in the absence of symptoms; concerns about testing; concern about testing positive and mistrust in the Government. There were more barriers than facilitators, however we did identify some facilitators to engaging in testing, including: desire to protect others; desire to return to normality; and hearing others’ positive experiences. We discuss barriers and facilitators in more detail below.

### Barriers to testing

For some, the absence of symptoms acted as a barrier to engaging in asymptomatic testing. This highlights a potential lack of understanding of asymptomatic infection and transmission, which has been highlighted in previous work [17]. In this context, individuals are using a model of infection where transmission is associated with the experience of symptoms. When associating infection with the experience of symptoms, there is no perceived need to test when symptoms are not present. Illness experience should be considered when developing and implementing policy, to ensure it can be enacted in everyday life [27]. For example, rather than focusing on asking people to test regularly even in the absence of symptoms, reframe messages to ask people to consider places and people they may have been exposed to that could result in them being infected, before they experience symptoms.

Parents also highlighted concerns about the process of testing. These included a concern that children would not be able to do the test well enough to provide accurate results, as well as general concerns about the accuracy and safety of the test. Concern about the accuracy of LFTs has been identified previously as a barrier to engaging in asymptomatic testing [17, 18], however our analysis identified that concerns about the accuracy of the tests were amplified when testing children because of concerns that the swab would not be performed to a certain standard. This was also highlighted in a previous study, where parents felt that self-administered tests reduced the accuracy of the results [28]. While there is specific guidance for testing young children [29], there is currently no specific guidance for older children with more autonomy, who may prefer to take the test themselves.

As well as concerns about the testing process, our analysis also highlighted concerns around the wider impact of children testing positive. This included potential financial implications of a child testing positive on other family members who may be unable to work. This is in line with previous research that has shown that a lack of available financial support for those self-isolating is a barrier to engaging with regular asymptomatic testing [18, 19].

In addition, the data highlighted that parents were absorbing the responsibility of their child testing positive, and they felt blamed by other parents for the impact of a positive test on other children in the class. This response could be due to the moralisation of health-related behaviours during Covid-19; those who did not adhere to public health guidance were perceived as irresponsible or careless [30]. Moralisation of health and illness can occur when health promotion focuses on provision of information and education campaigns to change behaviour [31]. Not only has this approach been shown to be ineffective [32], but it also reinforces the belief that individuals are solely responsible for their behaviour and negates any social or structural influences. This in turn can result in people experiencing stigma, as misconceived beliefs that individuals are to blame for their ill health are reinforced [31]. Parental concern over their child experiencing stigma because of school-based testing has been highlighted as a potential barrier to testing in schools [33], highlighting the importance of ensuring engaging parents in schools testing is not solely based on education campaigns and the provision of information on the testing offer.

The results also identified that for some, there was the belief that the schools testing policy was being used by the Government and media as a tool for controlling public behaviour through maintaining fear about Covid-19. Mistrust in government has been identified previously as a barrier to engaging in regular testing [18, 19] as well as other protective behaviours [34, 35]. Enhancing trust and legitimacy of government actions through open and honest communication has been shown to increase adherence to public health measures [36], therefore communicating openly about the schools testing policy, such as why it was introduced and how it will help reduce Covid-19 transmission may improve public support. There were also several comments indicating more extreme distrust in the Government, for example those comments that equated the schools testing policy to a form of abuse. While this was a minority few, the strength of the language used (e.g. “assault”, “grooming”) is indicative of strong beliefs.

### Facilitators of testing

For many parents there was a strong desire for schools to remain open, primarily because of the impact of school closures on learning and development and on children’s mental health. Concern over the impact of school closures on mental health and wellbeing of children has been identified previously [13], as has the potential for school closures to result in an increase in child abuse and neglect within homes [37]. This highlights the complex decisions parents are required to make, balancing risk of infection with impact on mental health and wellbeing.

Our analysis also highlighted the positive impact of sharing good experiences of testing. Sharing positive experiences has previously been identified as a facilitator to engaging in mass asymptomatic testing [19], therefore forums such as Mumsnet that give parents a safe space to share concerns and experiences may be useful resources for engaging parents when new policies are introduced. Our study focused on a limited time frame around the announcement of the schools testing programme, specifically to capture the public responses to testing being introduced in schools. As people become more accustomed to testing guidance, and the testing landscape changes, responses to regular testing will evolve. For example, one study of over 2000 secondary school students in England conducted in May 2021 (two months after the schools testing programme was implemented) found that most students felt comfortable with the home testing process and reported high levels of adherence to the testing programme [38]. There is currently no requirement to regularly test for Covid-19 in England, and free lateral flow tests are no longer available, however recent studies indicate intention to test appears to be relatively high [39, 40], although these studies do not focus specifically on testing in schools.

### Limitations and next steps

The analysis of social and online media data is a useful method of rapidly accessing public narratives on the schools testing policy. Further, analysis of social media data provides an insight into national narratives and perspectives on public health policies and can provide us with unfiltered opinions we may not have access to when using other evaluation methods such as surveys and interviews. However, our research does have some limitations. First, the demographic of social media users is different from the general population [41] and previous research has identified people who use social media have lower trust in government [35]. In addition, demographic characteristics vary depending on which social media platform is used [22]. We sampled data from a range of different social and online media sources to account for these demographic differences, however our sample is not representative of the wider population. Second, although we sampled parenting forums as well as online newspaper comments and social media, we do not know if comments were made by parents and teachers or other members of the general public. Further qualitative research with parents and teachers would help clarify some of the key themes we have identified in this study. Our study was limited to assessing perceptions during a short time when the schools testing programme was announced. Perceptions may have changed over time, however understanding the public response to the introduction of a new policy is important as it provides vital information for when new public health policies are introduced in future.

### Recommendations

Based on our findings, we recommend that to increase engagement with schools testing programmes in future, policy makers should: (1) communicate openly and honestly about the purpose of introducing testing in schools, including the reasoning behind it and how long this policy is intended to last – this will enhance the legitimacy of the policy; (2) provide information on the efficacy of using tests to help control the pandemic, including information about the accuracy of the tests and their role in reducing asymptomatic spread; (3) provide clear instructions for how to conduct a test, specifically on a child, including visual demonstrations such as videos; (4) consider having trusted communicators provide information directly to parents, facilitated by the schools – this may help to mitigate any mistrust in government; (5) acknowledge that some children with existing health conditions may find the testing process particularly challenging and therefore parents may need additional support to conduct the test; (6) facilitate online school-specific platforms for parents to share their experiences of testing – this may help parents to share their experiences, and enable concerns to be addressed; (7) provide information about isolation support payments and eligibility, and clearly signpost to these alongside testing information – this will give those who test positive the reassurance that financial help is available.

## Conclusion

Our analysis provides an insight into the national narratives around the schools testing policy and identifies several barriers and facilitators to engaging with testing in schools. While concerns about accuracy of asymptomatic testing and the need for adequate financial and social support are now well established, our analysis identified additional barriers around the testing process and impact of testing positive that were more challenging when children were involved. In addition, parents were often having to make complex decisions around engaging in testing, balancing theirs and their children’s apprehension about testing with concerns about missed learning. Alongside existing support measures, additional practical and social support for parents would be beneficial to facilitate parental engagement in school testing policies, for Covid-19 and other respiratory infections.

## Data Availability

The datasets analysed are available from the corresponding author on reasonable request.

## Abbreviations

LFT: lateral flow test
PCR: polymerase chain reaction tests
NPIs: non-pharmaceutical interventions
